# High Tibial Osteotomy (HTO), Unicompartmental Knee Arthroplasty (UKA), and Proximal Fibular Osteotomy (PFO) for Medial-Compartment Knee Osteoarthritis: A Narrative Review of Comparative Mechanisms, Clinical Outcomes, and Decision-Making

**DOI:** 10.3390/jcm14217882

**Published:** 2025-11-06

**Authors:** Furkan Yapıcı

**Affiliations:** Department of Orthopedics and Traumatology, Erzincan University Faculty of Medicine, 24100 Erzincan, Turkey; furkanyapici@hotmail.com

**Keywords:** high tibial osteotomy, unicompartmental knee arthroplasty, proximal fibular osteotomy, varus malalignment, medial knee osteoarthritis, survivorship

## Abstract

**Background:** Medial-compartment knee osteoarthritis with varus alignment is treated surgically by realignment (high tibial osteotomy, HTO), resurfacing (unicompartmental knee arthroplasty, UKA), or proximal fibular osteotomy (PFO), which aims to indirectly unload the medial tibial plateau. **Methods:** We conducted a structured narrative review (PubMed/MEDLINE, Google Scholar; 2000–2025; last search 30 August 2025) of comparative clinical, biomechanical and safety data for HTO, UKA and PFO, including prior meta-analyses and mechanistic reports. One hundred fourteen studies met prespecified criteria. **Results:** HTO reliably corrects coronal alignment and unloads the medial compartment; long-term survivorship varies by selection and technique, and complications include hinge fracture, delayed/nonunion and hardware problems. UKA typically yields faster early pain relief and recovery in pooled analyses, with implant-specific failure risks and mid-term revision dependent on design and surgical experience. PFO cohorts consistently report early pain and function gains with plausible biomechanical rationale, but evidence is dominated by small, heterogeneous series with short follow-up and limited comparative data. Adjusted head-to-head comparisons generally favor UKA for early pain yet show HTO and UKA can achieve similar patient-reported improvements in selected younger cohorts; robust comparative trials including PFO are lacking. **Conclusions:** HTO and UKA are established, mechanistically distinct options best matched to patient age, alignment, activity goals, and comorbidity. PFO is a low-burden, promising alternative with uncertain durability; longer-term, controlled evaluation and registry surveillance are required before broad adoption. Findings should inform shared decision-making while acknowledging differences in evidence maturity.

## 1. Introduction

Knee osteoarthritis (KOA) is a leading cause of pain, functional limitation, and healthcare utilization worldwide; prevalence increases with age, and the medial tibiofemoral compartment is the most frequently involved site, especially in varus-aligned knees and in populations with a high prevalence of varus morphology [1,2]. Mechanical malalignment—most commonly varus deformity—produces eccentric load transfer across the tibial plateau that accelerates cartilage degeneration and functional decline in the medial compartment; this biomechanical linkage provides the rationale for surgical strategies that restore alignment or selectively treat the diseased compartment [3,4].

Surgical options for symptomatic, isolated medial-compartment KOA therefore target one or more of the following goals: mechanical realignment and load redistribution (joint-preserving osteotomy), removal/replacement of the damaged articular surface (unicompartmental arthroplasty), or, increasingly, less-invasive decompressive procedures that seek to alter tibial–fibular load-sharing (proximal fibular osteotomy (PFO)). Historically, high tibial osteotomy (HTO) has been the principal joint-preserving procedure, whereas unicompartmental knee arthroplasty (UKA) has been the principal compartment-replacing option; more recently, proximal fibular osteotomy (PFO) has been advanced as a simpler and lower-cost alternative in selected patients [5,6,7]. The evolution of osteotomy around the knee—from 19th-century descriptions through modern opening-wedge and closing-wedge techniques and computer-assisted planning—frames contemporary choices between preservation and replacement [4,8]

High tibial osteotomy (HTO) is a deliberate coronal-plane realignment procedure that shifts the mechanical axis laterally to unload the medial compartment and thereby redistribute contact stresses; both medial opening-wedge and lateral closing-wedge techniques are established, and surgical planning must account for coronal correction as well as sagittal-plane consequences such as posterior tibial slope and patellar height [3,4,9,10]. In experienced hands, HTO can provide durable symptom relief and delay the need for arthroplasty, but long-term survivorship is variable across series and depends on patient selection, technique, and postoperative course [1,11]. Complications that affect outcomes after HTO include lateral-hinge fracture, nonunion or delayed union, infection, hardware problems, and loss of correction; perioperative and longer-term morbidity have been quantified in contemporary systematic assessments [12,13,14].

Unicompartmental knee arthroplasty (UKA) replaces only the diseased medial compartment, preserves bone stock and (in most designs) the cruciate ligaments, and is associated with faster early recovery and lower immediate morbidity compared with total knee arthroplasty; however, UKA carries specific implant-related failure modes, variable mid-term revision rates, and outcome trade-offs compared with HTO depending on the endpoint studied [15,16,17]. Comparative syntheses report that UKA often provides superior pain relief in the short to mid-term, while HTO may preserve or restore a greater range of motion and may be preferred for younger, highly active patients; return-to-activity outcomes and revision risks are influenced by implant type, bearing design, and surgical expertise [16,18].

Direct comparisons of HTO and UKA in the literature are heterogeneous with respect to indication, patient age, radiographic osteoarthritis grade, and outcome definitions, and meta-analyses have produced inconsistent recommendations. Some pooled analyses and individual studies favor UKA for symptomatic relief and early function, whereas adjusted comparative analyses indicate that, in selected cohorts (for example, younger patients with advanced Kellgren–Lawrence grades), HTO and UKA may yield comparable patient-reported outcomes when confounders are considered [16,19,20]. Thus, HTO is joint-preserving realignment that offloads the medial compartment by correcting varus (and tibial slope/patellar height), suiting younger, active patients with correctable malalignment. UKA resurfaces only the medial compartment with little global axis change, typically giving faster early pain relief and rehab but carrying implant-specific risks and requiring strict indications (intact ACL/MCL, limited deformity).

Proximal fibular osteotomy (PFO) has emerged as a distinct, minimally invasive approach that aims—by partial resection of the proximal fibula—to alter the mechanical support of the lateral tibial plateau and thereby decompress the overloaded medial compartment. Biomechanical rationales and finite-element models, together with radiographic and pilot clinical series, report immediate pain relief, functional improvement, and radiographic evidence of tibial plateau “settlement” or altered load distribution after PFO; nevertheless, the exact mechanistic pathway and the reproducibility of these changes remain subjects of active investigation [7,21,22]. Several single-center series and small prospective cohorts report favorable short-term symptom and function outcomes after PFO, and proponents have emphasized the low technical complexity and reduced cost compared with HTO or UKA [7,23,24].

At the same time, systematic reviews and the few comparative meta-analyses that include PFO underscore substantive limitations in the current evidence base: small samples, short follow-up, heterogeneity in surgical technique and outcome reporting, and a paucity of randomized or well-controlled prospective trials limit inference about durability, generalizability, and safety compared with established procedures [21,25,26]. Early comparative reports between PFO and HTO/UKA show mixed signals on magnitude and longevity of benefit, radiographic correction, and complication profiles, and therefore do not yet permit definitive recommendation of PFO as a routine alternative to HTO or UKA outside carefully selected indications or research settings [25,26,27].

Given the divergent invasiveness, biomechanical rationales, complication spectrums, cost implications, and maturity of evidence among HTO, UKA, and PFO, a structured narrative review is timely to synthesize mechanistic data, comparative clinical outcomes, complication profiles, patient-selection criteria, and gaps that should guide clinicians and investigators. This review examines the biomechanical basis, surgical techniques, reported efficacy and harms, and outstanding research priorities for HTO, UKA, and PFO in the management of medial-compartment knee osteoarthritis, with the explicit objective of informing patient-centered decision-making and defining priorities for high-quality comparative studies [19,21,25].

**Objective.** To provide a mechanism-informed, clinically oriented synthesis of HTO, UKA, and PFO for medial-compartment knee OA, integrating head-to-head comparisons where available, and explicitly grading the strength and limitations of evidence.

**Scope.** We emphasize comparative clinical outcomes (pain/PROs, ROM, survivorship), complication spectra, and selection signals by age, alignment, and disease severity, while critically appraising the immature PFO literature.

## 2. Methods

### 2.1. Design and Rationale

This narrative review synthesized contemporary evidence on high tibial osteotomy (HTO), unicompartmental knee arthroplasty (UKA), and proximal fibular osteotomy (PFO) for medial-compartment knee osteoarthritis. A fully systematic review was not pursued because of heterogeneous study designs and outcomes and the relatively immature evidence base for PFO compared with HTO/UKA. The aim was to provide a comprehensive, mechanism-informed overview to support clinical decision-making and to identify research gaps.

### 2.2. Eligibility Criteria

Systematic reviews/meta-analyses, randomized or non-randomized comparative studies, prospective or retrospective cohorts, and case series reporting clinical or radiographic outcomes, survivorship/revision, complications, return-to-activity, or resource signals for HTO, UKA, or PFO in adults with medial-compartment osteoarthritis/varus malalignment were included. Duplicates, non-English articles, conference abstracts without full text, and cadaveric/animal studies were excluded unless biomechanical insights were directly relevant to clinical endpoints. Eligibility rules were specified a priori. Data extraction for key endpoints was cross-checked to mitigate single-reviewer bias.

### 2.3. Information Sources and Search Window

PubMed/MEDLINE were searched for studies published between 1 January 2000 and 30 August 2025, to capture modern implant/technique eras for HTO/UKA and the emergence of PFO literature. During this PubMed/MEDLINE search, it became clear that most articles on PFO were found in the gray literature. Therefore, a Google Scholar search was also required to obtain these peer-reviewed articles that may not surface with strict PubMed indexing/field tags. Preprints or unpublished reports were not included. Also, although the formal search window was 2000–2025, seminal pre-2000 primary or technique papers and historically important reports (e.g., Coventry; early HTO/UKA methodology) were a priori deemed eligible for inclusion when they directly informed/guided contemporary practice.

### 2.4. Search Strategy

Boolean strings combined controlled vocabulary and free text. Example PubMed queries (adapted per database syntax):

- (“high tibial osteotomy” OR HTO OR osteotomy[tiab]) AND (medial[tiab] OR varus[tiab]) AND (knee[tiab])
- (“unicompartmental knee arthroplasty” OR UKA OR “partial knee”) AND (medial[tiab] OR varus[tiab])
- (“proximal fibular osteotomy” OR PFO) AND (knee[tiab] OR osteoarthritis[tiab])

### 2.5. Study Selection and Data Extraction

Records were deduplicated across databases and screened in two stages (title/abstract, then full text) against the prespecified criteria. The review was conducted by a single author. Screening occurred during September–October 2025. From each included study, data were abstracted on study design, country/setting, sample size, demographics, osteoarthritis grade, alignment parameters (e.g., HKA/FTA, JLCA, posterior tibial slope, and—where available—patellar-height indices), technique details (OWHTO/CWHTO; UKA bearing; PFO level), follow-up, primary outcomes (pain/PROs, function, radiographic correction), complications (with definitions), and reoperation/TKA conversion.

### 2.6. Registration

No prospective registration (e.g., PROSPERO) was filed because the intent was narrative and clinic-facing.

### 2.7. Yield

**Figure 1:**
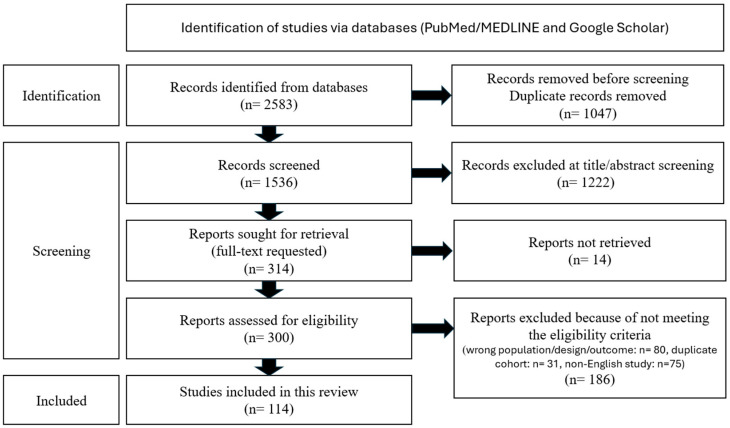
**PRISMA-style flow diagram of study selection.** Included studies: Systematic reviews/meta-analyses (n = 19); RCTs (n = 2); comparative cohorts (n = 30); clinical case series and mechanistic studies (finite-element/animal/technical with primary data) (n = 63); total = 114 studies.

The search identified 2583 records overall; following deduplication and exclusion, 114 studies met the inclusion criteria and were synthesized. Of these 114 studies, 1 is LOE I, 21 are LOE II, 28 are LOE III, 38 are LOE IV, and 26 are LOE V; 28 compare UKA vs. HTO, 4 compare HTO vs. PFO, 28 are various HTO studies, 9 are various UKA studies, and 35 are various PFO studies (LOE = level of evidence). All included articles were compiled into a Master Table S1 and are presented as Supplementary Materials at the end of the study. The List of Abbreviations is provided before the References. A PRISMA-style flow diagram of study selection (1 January 2000–30 August 2025; last search 30 August 2025) is presented as Figure 1. Indications/contraindications and selection signals for HTO, UKA, and PFO for medial-compartment knee OA are presented in Table S2, and key complications by procedure are presented in Table S3.

## 3. Discussion

### 3.1. Overview of the Evidence Base and Study Designs

Across high tibial osteotomy (HTO), unicondylar (medial) knee arthroplasty (UKA), and proximal fibular osteotomy (PFO), study designs remain heterogeneous, with relatively few randomized trials and many single-center observational series. For HTO vs. UKA, multiple meta-analyses consistently note selection differences (age, activity, Kellgren–Lawrence [KL] grade), variable techniques (opening-wedge vs. closing-wedge HTO; fixed vs. mobile-bearing UKA), and short-to-mid-term follow-up in much of the comparative literature [16,28,29,30]. For PFO, the bulk of publications are small prospective cohorts and case series; the largest syntheses emphasize short follow-up, inconsistent radiographic reporting, and potential small-study and geographic bias [31,32]. These features limit causal inference and generalizability across systems and surgeon volumes.

Brouwer et al.’s Cochrane review underscored that while valgus HTO improves pain and function, high-quality head-to-head comparisons (versus UKA or nonoperative care) remain scarce—reinforcing the heterogeneity and limited trial depth in this space [33]. In a meta-analysis, Gandhi et al. reported UKA was more likely than HTO to yield good/excellent results (with a trend toward better survivorship), whereas gait velocity was similar—again illustrating that cross-study differences in indications and technique color pooled estimates [34]. Current-concepts syntheses by Dettoni et al. and Rodríguez-Merchán highlight modest advantages for UKA in pain/function in some series but emphasize that neither UKA nor HTO is categorically superior when indications are respected [35,36]. Early comparative cohorts by Broughton et al. and Weale et al. favored UKA over HTO on proportions of good outcomes and long-term revision burden, but these historical series also reflect older implant eras and (often) closing-wedge techniques [37,38].

### 3.2. High Tibial Osteotomy (HTO): Durability, Correction Fidelity, and Safety Profile

#### 3.2.1. Durability

Long-term series confirm that appropriately indicated HTO can deliver durable symptom relief and defer total knee arthroplasty (TKA) for many years. In a contemporary 20-year closing-wedge cohort, Constantin et al. reported survivorship (failure = arthroplasty or revision HTO) of 88% at 5 years, 77% at 10 years, 63% at 15 years, and 44% at 20 years; in pre-defined “ideal candidates” (<55 years, BMI < 30, lower baseline pain), 20-year survivorship reached 62%, with KOOS Pain ≈ 91 and 97% satisfaction among survivors [11]. Classic large cohorts by Hui et al. and Flecher et al. likewise show ~79–80% 10-year and ~54–85% 15–20-year survival depending on case mix and technique [1,2]. Survivorship is selection-sensitive: age ≥ 55 and BMI ≥ 30 approximately double failure hazards in multivariable models [11]. In young–middle-aged adults, OWHTO corrects malalignment with meaningful short-term clinical gains and low early failure (~6%); posterior tibial slope (PTS) rises modestly, and most complications are minor (e.g., hardware irritation) [39]. Long-horizon cohorts from Coventry et al. and Naudie et al. similarly report ~75–90% 5–10-year survivorship, with younger age, better preoperative motion, and maintenance of valgus correction as key determinants [40,41]. Technique-driven series (e.g., Billings et al.) document excellent knee scores and low major complications after calibrated closing-wedge HTO with straightforward conversion to TKA when needed, whereas earlier work (e.g., Berman et al.) demonstrates gradual functional decline over 10–15 years, underscoring the importance of precise correction and selection [42,43]. After MOWHTO, survivorship was ~99% at 5 years and ~76% at 7.5 years, with older age and limited postoperative flexion predicting inferior outcomes; most complications were minor and manageable [44]. In Yapici et al. (504 MOWHTOs), 10-year survivorship was ≈ 95% despite many minor events and few surgery-requiring complications (~3%); planned correction was achieved in ~75%, highlighting alignment accuracy as a driver of longevity [45].

Hence, HTO can postpone TKA for a decade or more; durability hinges on patient selection (younger age, lower BMI, better pre-op function) and accurate valgus maintenance.

#### 3.2.2. Correction Fidelity and Sagittal Control (PTS/Patellofemoral Effects)

HTO’s core strength is deliberate coronal realignment and medial unloading, with predictable sagittal and patellofemoral consequences. Technique papers by Noyes et al. (3-Triangle method) and cadaveric/biomechanical work by Rodner et al. show how gap orientation and plate position modulate PTS (e.g., anterior opening or anteromedial plate placement increases PTS) [10,46]. Clinical series by Hinterwimmer et al. demonstrate that posteriorly placed spreaders and plates, complete posterior release, and maintaining an anterior gap ≈ ½ the posteromedial gap can keep PTS and patellar height stable after OW-HTO [9]. Narrative/technical reviews by Pullen et al. and Corgiat-Loia et al. quantify typical PTS changes (+~2° after OW-HTO; −~2° after CW-HTO) and propose pragmatic targets—WBL ≈ 62% lateral, KJLO < 4–6°, MPTA < ~95°—that correlate with durable outcomes [35,47]. Navigation-assisted HTO improves coronal precision and constrains unintended PTS change: Bae et al. linked mid-/long-term durability primarily to achieving the planned postoperative FTA, and Gebhard et al. reported a mean 1.5° deviation from the planned mechanical axis with most cases within ±3°, supporting navigation as a reliable “intraoperative ruler” [48,49].

Hence, fidelity of coronal correction and control of PTS/patellofemoral parameters are central to HTO success; navigation and disciplined gap/plate strategy reduce outliers.

#### 3.2.3. Safety Profile

Safety signals are well-characterized. In a 71-study review of 7836 patients, Miltenberg et al. estimated intra-operative LHF ≈ 9.1% in opening-wedge HTO, peroneal nerve injury ≈ 3.2% in laterally based HTOs, and postoperative complications ≈ 6.9% (deep infection ≈ 0.7%); reoperation is common but often minor (hardware removal ≈ 10%) [14]. A large single-center series by Martin et al. found only Class-3 (severe) events transiently depressed WOMAC at 6 months, with differences resolving by 12–24 months [13]. A high-discrimination risk model from Choi et al. (AUC 0.892) identified modifiable LHF predictors—e.g., posterior gap height, lateral fragment thickness, and hinge geometry—that translate directly into preventive tactics [50].

Hence, serious complications after HTO are uncommon; hinge integrity, sagittal control, and precise alignment are the levers that reduce risk and protect outcomes.

### 3.3. Unicondylar Knee Arthroplasty (UKA): Early Gains, Revision Modes, and Volume/İmplant Effects

#### 3.3.1. Classic İndications and Contemporary Survivorship

Classic indications synthesized by Kozinn et al.—elderly, non-obese, low-demand patients with intact cruciates and isolated compartment disease—help explain why strictly selected UKA cohorts often show faster rehabilitation and fewer early medical events than osteotomy or TKA [51]. Modern overviews report single-center survivorship ≈94–98% at 10–15 years in expert hands and emphasize that conversion to TKA is typically straightforward with primary components, though failure spectra differ by bearing design [52].

#### 3.3.2. UKA—Early Pain Relief

Compared with HTO, pooled comparisons consistently show faster early symptom improvement after UKA; however, the magnitude of short-term advantages is generally small and frequently below MCID thresholds. Meta-analyses by Han et al. [16] and Huang et al. [29] found less postoperative pain and fewer complications with UKA, while HTO tended to preserve greater ROM (e.g., +8.6° in Han et al. [16]; ~5–10° across reviews). Prospective comparative data likewise show better 24-month OKS, pain, EQ-5D, and satisfaction with UKA but sub-MCID mean differences [19]. Taken together, indications are that UKA appears to deliver earlier pain relief and faster short-term recovery with small, often sub-MCID average effects, whereas HTO tends to yield slightly greater ROM [16,19,28,29,30].

#### 3.3.3. UKA—Registries and Real-World Revision Burden

Signals diverge across national registries and claim analyses, consistent with confounding by indication, implant era/design, and volume effects. A Korean nationwide IPTW analysis reported higher conversion to TKA after HTO vs. UKA with excellent 8-year survivorship for both (≈96.8% UKA, 95.1% HTO) [53]. Conversely, a U.S. propensity-matched claims study found higher and earlier TKA after UKA at 5–10 years, while HTO carried slightly higher 1-year medical/mechanical complications [54]. Young-adult database work (≤55 years) shows greater outpatient feasibility, shorter OR time, and fewer 30-day events with UKA than HTO [55]. These discrepancies likely reflect differences in selection (age/activity/KL grade), coding of minor reoperations (e.g., plate removal after HTO), surgeon/hospital volumes, implant era, and rehabilitation protocols. Accordingly, we present revision risk as context-dependent rather than procedure-inherent [53,54,55].

#### 3.3.4. UKA—Cost-Effectiveness and Resource Use

Formal cost-utility trials within shared indications remain scarce, but resource signals generally favor UKA in recovery-sensitive patients: contemporary series support safe outpatient UKA with very low 90-day serious-event rates [56], whereas HTO commonly entails longer operative times, hardware costs, and frequent—but minor—secondary procedures (e.g., plate removal). For completeness, PFO is a low-burden day-surgery alternative with shorter OR time, less blood loss, and shorter stays than HTO in pooled analyses, though long-term durability and revision pathways are not yet defined. These considerations should be weighed alongside patient age, deformity origin, activity level, and goals.

#### 3.3.5. Failure Modes, Bearing Design, and Learning Curve

Failure patterns are implant-specific and volume-sensitive. Large series catalog dominant modes—bearing dislocation in mobile-bearing designs and aseptic loosening in fixed-bearing implants—with many events clustering ~4–5 years post-op [57,58]. Early experience concentrates complications during the learning curve (e.g., first ~30 procedures; [59]), and system-level comparisons suggest similar overall reoperation burden (~1.38 reoperations/100 component-years) but different spectra by bearing design [60]. Short-horizon safety is excellent: in 1000 consecutive UKAs, 90-day mortality was 0%, DVT 0.1%, and serious medical events ≤ 0.31% [59,61]. In very elderly candidates (≥85 years), UKA shows fewer early medical complications than TKA with comparable short-term function and survivorship [62].

#### 3.3.6. Limitations/Heterogeneity Note

Between-study differences in selection (older/less active for UKA; larger deformities to HTO), implant era/design (mobile vs. fixed bearing), surgeon/hospital volume, and coding of reoperations plausibly drive the registry-level discrepancies and temper cross-system generalization. We therefore report effect sizes (and MCID context) and present revision risk as sensitive to patient selection and center factors, not purely to procedure choice.

### 3.4. Proximal Fibular Osteotomy (PFO): Hypothesized Mechanism, Early Clinical Signal, and Heterogeneity

Mechanistically, PFO likely reduces medial load by weakening the fibular “lateral strut”, allowing relative lateral plateau settlement and load redistribution [22]. Finite-element models by Unal et al. [63] (≈26% reduction in medial contact pressure with lateral load shift), Kang et al. [64] (≈17–20% reduction in medial tibial cartilage stress across cut levels), and a mouse DMM model by Wang et al. [65] (attenuated subchondral sclerosis and lower OARSI scores with PFO) support plausibility; Qin et al.’s [22] prospective human study linked better outcomes to greater distal migration of the proximal fibula and lower BMI. After vascularized fibula graft harvest, the donor limb exhibited a small (~1.2°) valgus HKA shift that was statistically significant but likely clinically negligible, while knee and ankle function remained high with low donor-site morbidity [66]. A focused narrative by Vaish et al. distilled early clinical reports and operative ‘safe corridors’ (typically a 6–10 cm level below the fibular head), noting consistent short-term symptom gains but emphasizing the predominantly Level-III/IV evidence and short follow-up [67].

Clinically, pooled short-term effects are large. In the most recent meta-analysis, Jiang et al. (21 studies; 1006 knees) found VAS improvement ≈ −4.25 points, functional Hedges’ g ≈ 2.41, and alignment changes (HKA +1.7°, FTA −3.8°, medial joint space + 2.66 mm) [31]. Complications were dominated by transient sensory change (~5.9%) and common peroneal palsy (~2.25%); full weight-bearing within 1–3 days was typical. A severity-stratified synthesis by Liang et al. (13 studies; 788 knees) showed comparable clinical gains across KL-grade strata, suggesting KL-insensitive benefit at least in the short term [68]. A small prospective series reported excellent or good early outcomes in >90% after PFO, with minimal transient nerve symptoms. The procedure appeared effective at 6 months for KL II–III disease [69]. In KL IV varus knees, PFO led to measurable valgus correction and large KOOS and OKS improvements at short term. The uncontrolled, small cohort warrants cautious interpretation [70]. Conversely, a prospective series by Huda et al. documented waning benefit between 6 and 12 months (WOMAC 29.4 → 73.4), underscoring heterogeneity and the need for longer follow-up [71]. Early comparative work reports similar or greater short-term pain relief than HTO with lower peri-operative burden, but durability remains unproven [72]. In a prospective cohort (38 knees), PFO produced marked early pain relief and functional gains with only modest coronal correction and rare, transient complications—supporting PFO as a safe, minimally invasive option in selected patients [73]. Representative series—Subash et al. (30 patients, ~2-year follow-up), Kumar et al. (60 cases, day-one full weight bearing), Yadav et al. (prospective 12-month cohort), Bansal et al. (72 cases, ~18 months), Gupta et al. (20 cases, immediate full weight bearing), and Zuber et al. (PFO plus arthroscopic debridement)—all reported large early reductions in pain and improvements in function with modest valgization and low major morbidity [74,75,76,77,78,79]. In a small 10-patient cohort, PFO produced early improvements in KSS and pain over the first months, but transient dorsum-foot numbness and extensor hallucis longus weakness were common—underscoring the small sample size and short follow-up [80]. PFO significantly reduced pain and improved WOMAC at short term, with minimal change in ROM. Transient superficial and common peroneal palsies occurred but resolved, emphasizing careful nerve handling [81]. Outside Asia, Monreal et al. described a small Spanish mini-series with clear early pain relief and medial:lateral joint-space ratio improvement, but the sample was extremely limited, reinforcing the need for multicenter validation [82].

A position piece by Hüttner et al. summarized cadaveric data suggesting ≈20% medial pressure reduction after PFO and advocated meticulous nerve-sparing technique, yet called for multicenter randomized trials and registry surveillance before broad adoption [83]. Conceptually, Łuczkiewicz et al. proposed that PFO may mitigate medial meniscal extrusion by shortening the knee lever arm and reducing the adduction moment, while Demirkıran’s finite-element work suggested that adding PFO to medial UKA can slightly reduce polyethylene-insert contact stresses—hypotheses that remain to be proven clinically [84,85].

In short, for PFO, short-term pain/PRO gains are consistent across small cohorts and pooled analyses, valgization is modest, and neurologic symptoms/complications are usually transient; durability beyond 12–24 months remains uncertain.

Interpretation of PFO results: Because most PFO reports are Level III–IV, short-term, and regionally concentrated, with no registry-level surveillance, durability beyond 1–2 years and comparative safety are undefined; therefore, PFO should be considered selective/adjunctive rather than a substitute for HTO/UKA where durable alignment correction or implant longevity is the principal objective.

### 3.5. Head-to-Head Comparative Effectiveness

HTO vs. UKA. The most reproducible signal is superior early pain relief/function with UKA but greater ROM with HTO. Meta-analyses by Han et al., Fu et al., Huang et al., Cao et al., Zhang et al., and an overview by Ping et al. all converge on this pattern, with small absolute differences that often fall below established MCIDs [16,17,28,29,30,86]. Importantly, Hoorntje et al. performed a single-center retrospective cohort study of prospectively collected data comparing medial opening-wedge high tibial osteotomy (HTO) with medial unicompartmental knee arthroplasty (UKA) in younger patients (50–60 years) with Kellgren–Lawrence (KL) grade ≥ 3 medial OA (2016–2019), analyzing 84 HTO (mean age 55.0 ± 3.0 years, 79% men, BMI 27.8 ± 3.4, 75% KL-3) versus 130 UKA (55.7 ± 2.8 years, 47% men, BMI 28.7 ± 4.0, 64% KL-4) with prospectively scheduled PROs at 6, 12, and 24 months (response ≥ 87% at each time point). HTO used biplanar OW-HTO with TomoFix and full weight bearing from week 2 (no grafts; plate removal in 73% at median 14.1 months), while UKA used fixed or cementless Oxford mobile-bearing with immediate full weight bearing (patient-specific instrumentation in 74%). The primary outcome, Oxford Knee Score (OKS), analyzed with linear mixed models adjusting for baseline PROs, KL grade and sex, was 2.5 points lower in HTO than in UKA over 24 months (95% CI 1.0–4.0; *p* = 0.001), but this difference was below the OKS MCID (5 points); men scored +2.3 OKS points vs. women (*p* = 0.001), and KL-3 trailed KL-4 by 1.6 points (*p* = 0.027). Secondary outcomes likewise favored UKA on average—NRS pain at rest/activity lower in UKA by 0.7 and 1.2 points, EQ-5D-5L higher by 0.051, satisfaction NRS higher by 1.0, and anchor pain/function/GPR better by 0.6/0.7/0.6 points, respectively (all *p* ≤ 0.001)—yet the absolute magnitudes again sat below established MCIDs (≈1.6/10 for pain NRS; 0.074 for EQ-5D). Pre-op OKS was higher in HTO (26.6 ± 8.0 vs. 23.9 ± 6.9; *p* = 0.010). Conversion to TKA occurred in 3/84 (3.6%) HTO vs. 1/130 (0.8%) UKA during follow-up; other recorded complications included deep infection (3 HTO, 1 UKA) and HTO fixation/nonunion issues (1/2 cases). The authors conclude that UKA yields statistically better pain, quality-of-life, satisfaction, and OKS trajectories at 6–24 months, but between-group differences are not clinically important, so HTO is not inferior from the patient perspective in appropriately selected 50–60-year-olds with KL 3–4 medial OA [19]. Across 89 long-term series, a meta-analysis found comparable survivorship to TKA conversion for HTO and UKA (~84–87% at 9–12 years) but superior mid-term clinical scores with UKA; the authors recommend HTO for younger, active patients and UKA for older, pain-focused candidates [87]. Randomized evidence is limited; in the longest RCT, Stukenborg-Colsman et al. found 7–10-year survivorship of 77% for UKA vs. 60% for HTO in older, mildly varus knees [88]. At ~5-year follow-up, a comparative cohort found similar HSS and KOOS ADL/Sport after OWHTO and UKA, but UKA showed better KOOS Pain, Symptoms, and knee-related QoL. Revisions were uncommon in both groups, while secondary plate removal was frequent after HTO [89]. Early comparative cohorts by Broughton et al. and Weale et al. favored UKA on proportions of good results and on long-term revision burden, though both datasets pre-date contemporary OW-HTO techniques and modern UKA instrumentation [37,38].

HTO vs. PFO. A 23-study meta-analysis by Wu et al. suggested broadly similar short-term clinical efficacy, with PFO offering markedly lower peri-operative burden (operative time −38.8 min; shorter stays; less blood loss) [25]. Real-world series by Bayrak et al. (96 patients) favored HTO for larger OKS and alignment gains at 1 year—especially in non-obese patients—while showing clinically meaningful improvement after PFO [26]. Mechanistically, HTO achieves larger and more predictable axis correction; PFO provides modest coronal change with symptomatic relief, often rapidly. In early-stage medial OA, Gultac et al. found both partial proximal fibular resection and OW-HTO improved pain and WOMAC at 12 months, with OW-HTO achieving larger mechanical correction, whereas PFO enabled immediate weight bearing and only transient superficial peroneal symptoms [90].

UKA vs. PFO. High-quality head-to-head data are essentially absent. Narrative syntheses by Shanmugasundaram et al. and Ashraf et al. caution against extrapolating equivalence; until mid-/long-term durability and revision risks are defined, PFO should not supplant UKA in candidates where implant longevity is a principal objective [21,91]. Contemporary narrative and current-concepts reviews by Kozinn et al., Feeley et al., and Dettoni et al. also caution that, in the absence of controlled UKA–PFO trials, selection should continue to hinge on classic anteromedial OA criteria for UKA and on deformity biology for HTO [51,92,93].

Structured synthesis and limits of inference. Taken together, head-to-head signals are consistent but small: UKA tends to deliver earlier pain relief and faster short-term recovery, while HTO preserves slightly greater ROM; these average advantages are frequently sub-MCID, so they should not be over-interpreted. Methodological heterogeneity across sources (mixing OW- vs. CW-HTO techniques, differing UKA bearing/implant eras, variable inclusion of plate removal as a “reoperation”, uneven rehabilitation/weight-bearing protocols, and selection/volume effects) limits certainty and explains discordant registry/claims results. A practical, patient-stratified readout is therefore (i) younger, high-activity patients (<55–60 years) with larger correctable varus or slope abnormalities and desire to defer arthroplasty → HTO (accepting hardware removal and slower early recovery); (ii) older, pain-focused candidates (≈60+ years) with classic anteromedial OA, intact ACL, deformity typically <10°, and minimal flexion contracture → UKA (favoring outpatient pathways and faster early gains); (iii) early-stage medial OA with minimal deformity and strong preference for low peri-operative burden → PFO may be considered as a symptom-relief option, but mid-/long-term durability and revision pathways remain insufficiently defined. Across strata, survivorship and revision risk appear context-dependent (selection, implant era, and volume) rather than procedure-inherent; shared decision-making should therefore weigh patient goals, deformity biology, and center expertise at least as heavily as pooled averages.

### 3.6. Biomechanics and Radiographic Consequences That Shape Outcomes

HTO’s triplanar geometry is central to efficacy. Planning frameworks by Brown et al. and Smith et al. (Fujisawa point ≈ 62% WBL) and technique data by Noyes et al. demonstrate how coronal correction, PTS control, and patellar height interact [8,46,94,95]. Over-correction has patellofemoral costs: Yoon et al. found that postoperative WBL ratio >~62% increased the odds of patellofemoral cartilage deterioration 2.7–2.9× with worse KOOS pain/symptoms/ADL [96]. Procedure–deformity matching matters: in “grey-zone” varus (5–10°), Park et al. reported that UKA on tibial-vara knees (HR 18.0) and HTO without tibial vara (HR 8.7) independently predicted deterioration—underscoring the need to identify the deformity origin (tibial vs. femoral/intra-articular) [97]. Sagittal strategies are powerful adjuncts: in PCL-deficient knees, Giffin et al. showed that increasing PTS via anterior opening-wedge can reduce posterior sag; in ACL-deficient knees, Rodner et al. cautioned that unintended PTS increase shifts peak pressure posteriorly [10,98]. In ACL-deficient anteromedial OA, UKA with concomitant ACL reconstruction yields outcomes comparable to UKA in ACL-intact knees. HTO ± ACL reconstruction remains a reasonable option for younger patients or when extra-articular deformity predominates, albeit with a higher complication burden when procedures are combined [99,100]. Seminal technique guidance from Coventry et al. and later reviews by Wright et al. reiterated that accurate long-leg planning, intentional mild valgus, and attention to patellar height/tibial slope are the principal levers linking geometric correction to durable outcomes [101,102]. Complementing this, Bae et al. demonstrated that computer-assisted HTO improves proximity to target alignment and limits unintended slope changes compared with conventional techniques, translating to more consistent radiographic inliers [103].

UKA resurfaces without primary global axis correction; disease progression elsewhere is uncommon in modern series but remains a recognized failure path, especially with malposition or expanded indications in high-demand patients, as highlighted by Vince et al. [104].

PFO produces radiographic “settlement” and modest joint-space rebalancing rather than large axis change; FE studies (Unal et al. [63], Kang et al. [64]) and clinical signals (Qin et al. [22]) suggest patient- and level-dependent unloading rather than a uniform mechanical effect.

### 3.7. Return to Work/Sport, Satisfaction, and Predictors

Both HTO and UKA enable meaningful return to activity, with nuanced differences. In a pooled analysis, Belsey et al. (401 HTO vs. 1622 UKA knees) found that UKA typically delivers a larger pre-to-post activity increase (e.g., Tegner/UCLA), whereas HTO cohorts are more active at baseline and post-op [18]. In matched patients > 50 years of age, Screpis et al. [105] observed higher Tegner activity after opening-wedge HTO from 6 months through >4 years (3.83 vs. 3.27), with similar Lysholm and pain scores; Koh et al. [106] identified distinct dissatisfaction risks: advanced disease (Ahlbäck ≥2) for HTO vs. younger age (<60 years) and greater varus (HKA ≥5°) for UKA. Satisfaction is high in well-selected long-term HTO survivors: Constantin et al. [11] reported 97% satisfied/very satisfied at 20 years.

In active men aged <60 years, Nagel et al. showed high maintenance of sports participation after closing-wedge HTO despite modest long-term activity plateaus and ~16% conversion to TKA around 7 years—highlighting that pre-operative activity is the best predictor of post-operative activity [107]. For younger, high-demand cohorts, Feeley et al. advocated an algorithm prioritizing HTO when varus malalignment drives symptoms and reserving UKA for strictly unicompartmental disease seeking faster recovery [92].

### 3.8. Complications, Reoperations, and Survivorship—What the Large Datasets Show

Registry- and claims-based comparisons are directionally mixed, reflecting case-mix and technique era. In a Korean nationwide IPTW analysis, Yoo et al. found higher risk of conversion to TKA after HTO vs. UKA (adjusted HR 1.42), with both procedures showing excellent 8-year survival (UKA 96.8% vs. HTO 95.1%) [53]. In a U.S. PearlDiver matched study, Serbin et al. reported the opposite pattern—higher and earlier TKA after UKA (5-year 7.67% vs. 3.36%; 10-year 9.16% vs. 4.49%)—alongside slightly higher 1-year medical/mechanical complications after HTO [54]. These discrepancies likely reflect differences in indication thresholds, implant eras (e.g., modern mobile- vs. fixed-bearing), rehabilitation protocols, and coding. Meta-analyses temper the signal: Zhang et al. [86] found similar overall revision risk but lower long-term revisions after HTO in >60-month follow-up subsets; Huang et al. [29] and Cao et al. [30] observed fewer complications (and, on sensitivity analysis, fewer revisions) with UKA. The message for practice is not that one procedure is categorically “safer”, but that surgeon expertise, implant selection, and indication discipline dominate outcomes. In young adults aged ≤ 55 years, Debopadhaya et al.’s NSQIP analysis showed UKA was performed far more often than HTO and was associated with shorter operative time, fewer 30-day complications and reoperations, and a greater likelihood of outpatient management—despite HTO patients being younger and ostensibly healthier [55].

Why estimates differ across registries/claims or why registries disagree:

(i) confounding by indication (older, less active patients often selected for UKA; alignment magnitude differs); (ii) technology era and bearing design (mobile vs. fixed, learning-curve effects); (iii) coding practices and capture of minor reoperations (e.g., plate removal after HTO); (iv) surgeon and hospital volumes; and (v) rehabilitation protocols. These factors plausibly explain opposing hazard signals across datasets and temper cross-system generalization.

Downstream TKA after prior HTO vs. prior UKA. A 10,045-patient meta-analysis by Li et al. found slightly better knee scores after TKA in patients with prior HTO (MD +3.35, below KSS MCID) and lower need for revision implants (OR 0.11), with similar complications and infection rates—pragmatically reassuring when joint preservation is staged toward arthroplasty [108].

### 3.9. Practicalities: Peri-Operative Burden, Learning Curve, and Health-System Context

Resource signals differ meaningfully. UKA can be safely performed as outpatient: Codyet al. showed similar 90-day outcomes in ambulatory surgery centers vs. hospital outpatient departments [56]. PFO is consistently “low-burden”: Wu et al.’s meta-analysis recorded ~39 min shorter operative time, markedly less blood loss, and ~3.8 days shorter stays than HTO, and most PFO series permit immediate full weight-bearing [25]. However, cost advantages should not eclipse uncertainty about durability and revision risk. Utilization has shifted toward UKA in U.S. claims: Nwachukwu et al. documented UKA growth (+4.7% CAGR, 2007–2011) versus HTO decline (−3.9%), a pattern likely driven by rapid recovery expectations and implant/technique maturation [109].

Historically informed current-concepts reviews by Coventry et al., Wright et al., and Mattei et al. also stress that learning-curve effects and technique choice (e.g., closing- vs. opening-wedge) drive complication spectra—peroneal-nerve risk and intra-articular fracture in some CW-HTO series versus nonunion/loss of correction concerns in OW-HTO—factors that should be weighed alongside resource and training constraints [101,110,111].

### 3.10. Patient Selection—Aligning Mechanics with Goals and Comorbidity

HTO fits younger, active patients with varus malalignment who prioritize joint preservation, are willing to accept staged recovery, and in whom deformity is tibial-based or coronal malalignment is a principal pain driver. Age and BMI strongly influence longevity (e.g., Trieb et al. [112]: 10-year survival ~90% if <65 years vs. 70% if ≥65 years; ≈7.6% higher failure risk per additional year). Over- or under-correction and patellofemoral status must be explicitly managed; avoid WBLR > ~62% to reduce PF cartilage deterioration (Yoon et al. [96]).

UKA suits older or recovery-sensitive patients who meet strict anteromedial OA criteria with intact cruciates/MCL and correctable deformity. Surgeon/implant experience is crucial to minimize dislocation/loosening. In younger/high-demand patients, broader indications remain debated (Vince et al. [104]).

PFO may be considered in medically frail patients or low-resource settings as a same-day, immediate-weight-bearing option. Counsel on neurologic risk (sensory ~6%, transient CPN ~2–3%) and uncertain durability; early data suggest BMI and proximal tibiofibular mechanics modulate outcomes (Qin et al. [22]), and adjunctive arthroscopy or subchondral drilling may augment short-term benefit (Irismetov et al. [113]; Karapınar et al. [114]). Until mid-/long-term comparative data accrue, we view PFO as selective/adjunctive rather than a replacement for HTO or UKA in patients who need durable alignment correction or implant longevity.

From a classic selection standpoint, Coventry et al. recommended HTO for physiologically young, active patients targeting ~3–5° mechanical valgus, whereas Kozinn et al. outlined UKA criteria (elderly, non-obese, intact cruciates, strictly unicompartmental disease) that remain useful for minimizing early failures [51,110].

Methodological caveats—power, publication bias, and technology-era confounding. Across these sections, several signals (e.g., early PRO advantages with UKA, ROM preservation with HTO, low-burden peri-op profile with PFO) should be interpreted with caution given (i) limited statistical power in many head-to-head cohorts (small samples, wide CIs, underpowered subgroup analyses by age, KL grade, or alignment severity); (ii) potential publication and reporting bias (single-center “expert” series, survivorship bias from loss to follow-up, selective reporting of favorable time points; meta-analytic funnel asymmetry cannot be excluded, and trim-and-fill would likely narrow effects); and (iii) confounding by technology era and learning curve (pre- vs. post-2015 implant iterations; mobile- vs. fixed-bearing UKA; navigation/PSI/robotic adoption; TomoFix OW-HTO vs. legacy techniques; evolving weight-bearing/rehab protocols). Where possible, we prioritized absolute effect sizes and MCID context, noted heterogeneity, and emphasized context-dependent revision/survivorship rather than procedure-inherent claims. Future work should pre-register protocols, ensure adequately powered multicenter designs, stratify by implant era and surgeon volume, and report patient-important outcomes with standardized thresholds for clinical significance.

### 3.11. Synthesis and Clinical Guidance

Pain/function (short term): UKA > HTO > PFO? UKA most consistently wins on early pain relief and many PROs, though absolute differences vs. HTO are often <MCID when indications are respected (Hoorntje et al. [19]). PFO also produces large early pain relief/PRO gains, but evidence quality is lower, and durability is unknown (Jiang et al. [31]).

Range of motion and mechanics: HTO typically preserves or improves ROM and—critically—corrects axis and can tune PTS/patellar height; UKA preserves native kinematics but does not correct global axis; PFO indirectly unloads the medial compartment with modest coronal change.

Complications/revision: UKA tends toward fewer early complications in pooled analyses, but design-specific failure modes demand vigilance. HTO complications are technique-dependent and largely preventable with meticulous planning; long-term revision risk is strongly selection-dependent. For PFO, neurologic issues are the main peri-operative risk signal; revision data remain sparse. Conflicting registry/claims findings (Yoo et al. [53] vs. Serbin et al. [54]) should be interpreted through indication and era lenses.

A pragmatic shared-decision framework is therefore as follows: choose HTO when mechanical axis correction is the main therapeutic lever and joint preservation is prized; choose UKA when isolated compartment disease with ligament competence is present and rapid recovery is prioritized; reserve PFO for selective scenarios (frail patients, resource-limited settings, or as an adjunct) with explicit counseling about uncertainties.

### 3.12. Gaps and Priorities for Future Research

Key gaps include (i) high-quality randomized or well-controlled prospective trials in “indication-overlap” patients (e.g., moderate varus KL 3–4), comparing modern OW-HTO vs. contemporary UKA; (ii) multicenter registries for PFO with standardized radiographic and PRO definitions, explicit adverse-event capture, and ≥5–10-year follow-up; (iii) imaging-based mechanistic studies clarifying how PFO changes proximal tibiofibular kinematics and knee adduction moment in vivo; and (iv) cost-effectiveness analyses that incorporate reoperation pathways (e.g., TKA after prior HTO vs. UKA). Echoing Brouwer et al.’s Cochrane appraisal, the field still needs randomized or rigorously controlled trials directly comparing modern OW-HTO and contemporary UKA within shared indications, plus multicenter registries and longer follow-up for PFO [33].

### 3.13. Limitations of This Narrative Review

This review is narrative by design and not a systematic or quasi-systematic review. Methods prioritized clinical overview rather than exhaustive reproducibility: searches were limited to PubMed/MEDLINE and Google Scholar, no PROSPERO registration was filed, screening and extraction were performed by a single reviewer, and Google Scholar was also screened to minimize retrieval bias; only peer-reviewed articles were included, and non-English reports were excluded. Accordingly, the synthesis is susceptible to the typical limitations of non-systematic approaches—selection bias in study inclusion, variable methodological quality across source studies, and an inability to quantitatively pool outcomes given heterogeneous designs and follow-up durations. Because the PFO literature is dominated by small, single-center studies from a limited set of regions, with follow-up typically ≤12–24 months and no registry-level surveillance, it is vulnerable to small-study, publication, selection, and language biases; accordingly, any estimates of comparative effectiveness or safety should be treated as low-certainty—qualitative, provisional, and primarily hypothesis-generating rather than definitive. Data extraction for key endpoints was cross-checked to mitigate single-reviewer bias.

## 4. Conclusions

HTO and UKA are both evidence-based options for medial-compartment knee osteoarthritis, with distinct mechanistic targets, trade-offs, and complication spectra; neither is categorically superior across all patients. UKA most reliably improves early pain/function; HTO most reliably corrects axis and preserves motion with selection-dependent long-term survivorship.

PFO is a promising, low-burden procedure that yields rapid symptomatic improvement in early series and offers KL-insensitive gains, but current evidence is insufficient to equate it with HTO or UKA where durable mechanics or implant longevity are paramount. PFO offers reproducible short-term symptom improvement in small, predominantly single-center series, with modest alignment change and transient neurologic risks; however, durability, comparative effectiveness and registry-level safety remain undefined. We therefore view PFO as selective/adjunctive pending longer-term, well-controlled studies.

UKA generally delivers faster early pain relief and functional recovery; HTO most reliably restores limb alignment and maintains knee motion with established mid- to long-term durability in selected patients; PFO provides a low-burden route to short-term symptom improvement but requires confirmation of long-term effectiveness and safety.

Procedure choice should be individualized to deformity origin, age/activity, OA grade, comorbidity, and local expertise, within a shared-decision framework that transparently communicates the strengths and uncertainties of each strategy.

Finally, it should be emphasized that, owing to the narrative design, the superiority of one method over another cannot be directly asserted; rather, only the existence of heterogeneous, unsystematic reports suggesting such patterns can be noted, and definitive conclusions cannot be drawn. 

## Data Availability

All extracted data are contained within the article and its Supplementary Materials.

## Supplementary Materials

The following supporting information can be downloaded at: https://www.mdpi.com/article/10.3390/jcm14217882/s1, Table S1: Master Table; Table S2: Indications/contraindications & selection signals for HTO, UKA, and PFO for medial-compartment knee OA; Table S3: Key complications by procedure.

## List of Abbreviations

ADL	Activities of Daily Living
AKSS	American Knee Society Score (often used interchangeably with KSS)
BMI	Body Mass Index
CAS	Computer-Assisted Surgery
CI	Confidence Interval
CPN	Common Peroneal Nerve
CWHTO	Closing-Wedge High Tibial Osteotomy (also written LCWHTO for “lateral” closing-wedge)
DMM	Destabilization of the Medial Meniscus (preclinical mouse OA model)
DVT	Deep Vein Thrombosis
EQ-5D	EuroQol-5 Dimensions Health Utility Index
FE/FEA	Finite-Element (Analysis)
FTA	Femorotibial Angle
HKA	Hip–Knee–Ankle Angle
HSS	Hospital for Special Surgery (knee) Score
HTO	High Tibial Osteotomy
HR	Hazard Ratio
IPTW	Inverse Probability of Treatment Weighting
JLCA	Joint Line Convergence Angle
JS/JSW	Joint Space/Joint Space Width
JSR	Joint Space Ratio (e.g., medial:lateral)
KL	Kellgren–Lawrence (radiographic osteoarthritis grading)
KOA	Knee Osteoarthritis
KOOS	Knee Injury and Osteoarthritis Outcome Score
KSS/KSFS	Knee Society Score/Knee Society Function Score
LDFA	Lateral Distal Femoral Angle
LOE	Level of Evidence
MCID	Minimal Clinically Important Difference
MD	Mean Difference (effect-size metric)
MCL	Medial Collateral Ligament
MOWHTO	Medial (Opening-Wedge) High Tibial Osteotomy
MPTA	Medial Proximal Tibial Angle
OA	Osteoarthritis
OKS	Oxford Knee Score
OR	Odds Ratio
OWHTO	Opening-Wedge High Tibial Osteotomy
PF/PFJ	Patellofemoral/Patellofemoral Joint
PFO	Proximal Fibular Osteotomy
PRO/PROs	Patient-Reported Outcome(s)
PSI	Patient-Specific Instrumentation (guides/templates)
PTS	Posterior Tibial Slope
QoL	Quality of Life
RCT	Randomized Controlled Trial
ROM	Range of Motion
TKA	Total Knee Arthroplasty
UCLA	University of California, Los Angeles Activity Score
UKA	Unicompartmental Knee Arthroplasty
WBL/WBLR	Weight-Bearing Line/Weight-Bearing-Line Ratio
