# Supplementary material for: High Tibial Osteotomy (HTO), Unicompartmental Knee Arthroplasty (UKA), and Proximal Fibular Osteotomy (PFO) for Medial-Compartment Knee Osteoarthritis: A Narrative Review of Comparative Mechanisms, Clinical Outcomes, and Decision-Making
Source: J Clin Med. 2025 Nov 6;14(21):7882. doi: 10.3390/jcm14217882 (PMC12608725; doi:10.3390/jcm14217882)
Supplement: Supplementary file 1 [file jcm-14-07882-s001.zip › jcm-3969040-supplementary.pdf]

Table S1. Master Table.

Author (Year)	Country	Design	LOE	Procedure	Key Outcomes
**Colsman et al.** (2001)	Germany	Prospective randomized trial 60 patients	II	UKA vs HTO	In an RCT of older patients, HTO and UKA achieved comparable KSS improvements, with greater ROM after HTO. Mid-term survivorship favored UKA, and HTO had more complications and earlier revisions.
**Gandhi et al.** (2009)	Canada	Systematic review & meta-analysis 6 studies	II	UKA vs HTO	A 2008 meta-analysis found UKA more likely to yield good/excellent results than HTO, with a trend toward better survivorship. Gait velocity was similar, while reported complications varied by procedure and study.
**Spahn et al.** (2013)	Germany	Meta-analysis 89 studies	II	UKA vs HTO	Pooled long-term series showed similar survivorship to TKA conversion for HTO and UKA (~84–87% at 9–12 years), with UKA scoring better clinically at mid-term. The authors recommend HTO for younger, active patients and UKA for older, pain-focused patients.
**Fu et al.** (2013)	China	Systematic review & meta-analysis 11 studies	II	UKA vs HTO	Across 11 studies, UKA achieved more excellent/good functional results than HTO, whereas HTO preserved greater postoperative ROM. Complications and revision risks were similar, with a trend to faster walking after UKA.
**Mancuso et al.** (2016)	Italy	Systematic review 26 studies	II	UKA vs HTO	In ACL-deficient unicompartmental OA, UKA combined with ACL reconstruction achieved outcomes comparable to UKA in ACL-intact knees. HTO ± ACLR is reasonable for younger or extra-articular deformity cases but carries a higher complication burden when combined.
**Han et al.** (2017)	South Korea	Systematic review & meta-analysis 16 studies	II	UKA vs HTO	Meta-analysis of 16 studies found UKA yielded better function, less pain, and fewer complications than HTO, while HTO preserved greater ROM. Overall revision risk was similar, though closing-wedge HTO carried higher revision risk than UKA, unlike opening-wedge HTO.
**Santoso et al.** (2017)	Indonesia	Systematic review & meta-analysis 15 studies	II	UKA vs HTO	Across 15 studies, UKA had better proportions of excellent/good results, less pain, and fewer complications than HTO, while HTO preserved more ROM. Functional scores and revision rates were similar overall, and opening-wedge HTO narrowed the performance gap.
**Cao et al.** (2018)	China	Systematic review & meta-analysis 10 studies	II	UKA vs HTO	Pooling 10 comparative studies, UKA had fewer complications and less postoperative pain, while HTO maintained greater ROM. Overall revision risk was similar, though sensitivity analyses suggested lower revisions with UKA.
**Belsey et al.** (2021)	UK	Systematic review with pooled activity outcomes 13 studies	II	UKA vs HTO	Return-to-activity improved after both HTO and UKA, with UKA showing a greater increase despite HTO patients being more active preoperatively. Clinically meaningful gains were achieved for Lysholm and OKS, while Tegner changes rarely met MCID.

Author (Year)	Country	Design	LOE	Procedure	Key Outcomes
**Huang et al.** (2022)	China	Systematic review & meta-analysis 29 studies	II	UKA vs HTO	In 29 comparative studies, UKA reduced complications and slightly improved Lysholm/HSS scores versus HTO, with similar pain and walking speed. ROM differences depended on implant design—fixed-bearing UKA lagged HTO, whereas mobile-bearing matched it—and sensitivity suggested higher revision with HTO.
**Zhang et al.** (2023)	China	Systematic review & meta-analysis 38 studies	II	UKA vs HTO	Across 38 studies, UKA produced less pain, fewer complications, and better WOMAC function, while HTO yielded slightly greater ROM. Revision rates were similar overall, though longer-term subgroups favored HTO; heterogeneity was substantial.
**Li et al.** (2025)	China	Systematic review & meta-analysis 11 studies	II	UKA vs HTO	Compared with TKA after prior UKA, TKA after prior HTO showed slightly better function and less need for revision implants, though differences were small. Overall complications and revision rates were similar, with sensitivity analyses favoring HTO-TKA for lower re-revision and shorter operative time.
**Broughton et al.** (1986)	UK	Retrospective comparative cohort 91patients	III	UKA vs HTO	Early comparative data favored UKA over HTO, with higher rates of good outcomes and fewer revisions at 5–10 years. UKA patients were more often pain-free at review.
**Weale et al.** (1994)	UK	Retrospective long-term matched cohorts 122 knees	III	UKA vs HTO	At 12–17 years, UKA maintained better clinical results and fewer revisions than HTO in matched cohorts. A higher proportion of UKA knees remained pain-free long term.
**Nwachukwu et al.** (2014)	USA	Retrospective claims utilization trends	III	UKA vs HTO	U.S. practice patterns 2007–2011 showed increasing UKA use and declining HTO, with UKA favored in older, female patients and HTO in younger, male cohorts. The UKA:HTO ratio rose markedly over time.
**Petersen et al.** (2016)	Germany	Retrospective comparative cohort 48 patients	III	UKA vs HTO	At ~5 years, OW-HTO and UKA had similar HSS and KOOS ADL/Sport scores, but UKA reported better KOOS Pain, Symptoms, and knee-related QoL. Revision rates were low and comparable, though HTO often required secondary plate removal.
**Koh et al.** (2019)	South Korea	Retrospective comparative cohort 241 patients	III	UKA vs HTO	With satisfactory alignment, UKA delivered superior 2-year WOMAC subscales and activity outcomes compared with HTO, despite HTO being used in younger, more varus knees. Dissatisfaction was associated with advanced OA for HTO and with younger age and greater varus for UKA.
**Yoo et al.** (2023)	South Korea	Nationwide IPTW claims cohort	III	UKA vs HTO	In a national cohort, HTO carried a higher adjusted risk of conversion to TKA than UKA, although absolute 8-year survivorship was high in both. Notably, TKA after prior UKA had a higher revision risk than TKA after prior HTO.

Author (Year)	Country	Design	LOE	Procedure	Key Outcomes
**Hoorntje et al.** (2023)	Netherlands	Retrospective single-centre comparative cohort 214 patients	III	UKA vs HTO	In 50–60-year-olds with medial KOA, UKA showed statistically better OKS, pain, EQ-5D, and satisfaction than HTO over 24 months, but differences were below MCIDs. Low conversion and complication rates in both groups suggest HTO remains a patient-relevant alternative when appropriately indicated.
**Serbin et al.** (2023)	USA	Retrospective propensity-matched claims cohort	III	UKA vs HTO	In U.S. claims–matched cohorts, UKA converted to TKA more frequently and earlier than HTO at 5–10 years. However, HTO had higher 1-year medical and surgical complications, informing trade-offs in procedure selection.
**Debopadhaya et al.** (2024)	USA	Retrospective database cohort 2318 patients	III	UKA vs HTO	Among young adults ≤55, UKA was performed far more often than HTO and was associated with shorter operative time, fewer 30-day complications, and fewer reoperations. HTO cases had longer hospital stays despite healthier baseline profiles.
**Park et al.** (2025)	South Korea	Retrospective comparative cohort 120 patients	III	UKA vs HTO	In moderate medial KOA with 5–10° varus, both HTO and UKA achieved meaningful improvement, but unsatisfactory alignment predicted mid-term deterioration. Matching procedure to deformity origin mattered: HTO performed without tibial varus and UKA in tibial-vara knees had worse outcomes.
**Kozinn et al.** (1989)	USA	Narrative current concepts review	V	UKA vs HTO	Classic indications for UKA include elderly, non-obese, low-demand patients with isolated compartment disease and intact cruciates. UKA offers faster rehab and fewer early complications than HTO/TKA but requires meticulous technique.
**Vince et al.** (2004)	USA	Narrative/editorial review	V	UKA vs HTO	This critique warns that extending UKA to younger, high-demand patients may increase complications despite excellent results in classic indications. Compared with HTO, UKA often yields better pain relief and recovery, but proper selection is crucial.
**Feeley et al.** (2010)	USA	Narrative review	V	UKA vs HTO	For active younger patients, nonoperative care remains first-line, with HTO favored for varus unicompartmental disease and UKA for isolated compartment disease seeking faster recovery. HTO shows durable survivorship in selected cohorts, while UKA offers high satisfaction and return-to-sport rates exceeding TKA.
**Dettoni et al.** (2010)	Italy	Narrative review	V	UKA vs HTO	Synthesis of head-to-head studies suggests UKA often has modest advantages in pain and survivorship, though many comparisons involve closing-wedge HTO. Neither procedure is definitively superior; selection should be individualized, with TKA as the common revision endpoint.

Author (Year)	Country	Design	LOE	Procedure	Key Outcomes
**Rodriguez-Merchán et al.** (2016)	Spain	Narrative current concepts review	V	UKA vs HTO	This review argues that UKA often provides better function, pain relief, and lower revision/complication rates, but HTO is preferred when axis correction or constitutional varus predominate. Both procedures succeed when indications and technique are respected.
**Ping et al.** (2022)	China	Systematic overview (umbrella) of meta-analyses	V	UKA vs HTO	Across 10 meta-analyses, UKA generally produced less pain and more excellent/good results, whereas HTO provided superior postoperative ROM. Differences in revision and complications were inconsistent, with closing-wedge HTO showing higher revision risk than UKA.
**Khalil et al.** (2021)	Egypt	Prospective randomized clinical trial 40 patients	II	HTO vs PFO	In a small RCT, PFO and OW-HTO both improved pain, function, and medial joint space, with PFO showing greater pain reduction at short term. Complication profiles differed—PFO had transient neuropraxia while HTO had delayed weight-bearing and one DVT.
**Wu et al.** (2022)	China	Systematic review & meta-analysis 23 studies	II	HTO vs PFO	Meta-analysis found PFO had clear perioperative advantages over HTO—shorter surgery, less blood loss, and shorter hospitalization—while delivering similar short-term pain and function. Complication rates were comparable, but trial quality and follow-up were limited.
**Bayrak et al.** (2024)	Türkiye	Retrospective comparative cohort 96 knees	III	HTO vs PFO	At 1 year, both PFO and HTO improved OKS, pain, and radiographic measures, with HTO yielding larger overall gains, especially in non-obese patients. In obese patients, clinical improvements were similar, though HTO preserved radiographic advantages.
**Gultac et al.** (2025)	Türkiye	Retrospective comparative cohort 90 patients	III	HTO vs PFO	In early medial OA, both PPFR and OWHTO improved pain and WOMAC at 12 months, with OWHTO achieving greater mechanical correction. PPFR enabled immediate weight-bearing and had only transient superficial peroneal symptoms.
**Brouwer et al.** (2007)	Netherlands	Cochrane systematic review 13 RCTs	I	HTO	Cochrane review found “silver-level” evidence that valgus HTO improves pain and function, but few robust comparisons to UKA or nonoperative care exist. Technique studies suggest trade-offs without a clearly superior method.
**Miltenberg et al.** (2024)	Netherlands	Systematic review 71 studies	II	MOWHTO	Across 7,836 HTOs, overall postoperative complications were ~7% and intraoperative events ~6%, with LHF a key OWHTO risk. Reoperation occurred in ~15% (mostly plate removal) and conversion to arthroplasty in ~12% at short- to mid-term follow-up.
**Berman et al.** (1991)	USA	Retrospective cohort 39 knees	III	HTO	In a historical cohort, HTO provided several years of pain relief but outcomes declined over 10–15 years, with ~23% converting to TKA. Better results occurred in younger patients with unicompartmental disease, <12° deformity, and ROM ≥90°.

Author (Year)	Country	Design	LOE	Procedure	Key Outcomes
**Coventry et al.** (1993)	USA	Retrospective long-term cohort 87 knees	III	HTO	Long-term survival after proximal tibial osteotomy was 89% at 5 years and 75% at 10, with best results when postoperative valgus ≥8° and patients were not overweight. Alignment was stable over time.
**Naudie et al.** (1999)	Canada	Retrospective ≥10-year survivorship cohort 106 knees	III	HTO	HTO survival decreased over two decades, with ~30% unrevised at 20 years; younger age and preop flexion ≥120° predicted better longevity. Lateral tibial thrust and delayed/nonunion were strong failure predictors.
**Trieb et al.** (2006)	Austria	Retrospective survival analysis 94 knees	III	HTO	Age strongly influenced HTO longevity: 10-year survival was ~90% in <65 versus ~70% in ≥65. Failures occurred earlier in older patients, suggesting HTO should be used cautiously beyond 65.
**Flecher et al.** (2006)	France	Retrospective long-term series 301 knees	III	LCWHTO	In 301 CW-HTOs with mean 18-year follow-up, survivorship reached ~85% at 20 years (any revision endpoint). Younger age, lower OA grade, BMI <30, and postoperative valgus >6° independently improved outcomes.
**Gstöttner Michaela et al.** (2008)	Austria	Retrospective long-term cohort 134 knees	III	LCWHTO	Long-term CW-HTO survivorship declined from ~94% at 5 years to ~54% at 18 years, with age independently worsening outcomes. Pain improved significantly; complications included thrombotic events and peroneal palsy.
**Bae et al.** (2009)	South Korea	Retrospective comparative cohort 100 knees	III	LCWHTO	Compared with conventional CWHTO, navigation improved accuracy, reduced variability, and minimized unintended posterior slope change. Radiographic targets were met more consistently with CAS.
**Bae et al.** (2016)	South Korea	Retrospective comparative cohort 150 knees	III	LCWHTO	At mid- to long-term follow-up, navigation produced better early knee scores and more alignment inliers than conventional CWHTO. Survivorship was high in both, and postoperative alignment (FTA >7° valgus) independently predicted durability.
**Yoon et al.** (2019)	South Korea	Retrospective cohort 135 knees	III	MOWHTO	After MOWHTO, excessive valgus (WBL ratio >~62%) was associated with patellofemoral cartilage deterioration and inferior PROs. Surgeons should avoid overcorrection to protect the PF joint.
**Choi et al.** (2021)	South Korea	Retrospective case-control 261 patients	III	MOWHTO	A large OW-HTO series identified geometric and osteotomy-configuration factors that predict lateral hinge fracture and proposed a risk score with high discrimination. LHF subtypes showed different alignment behaviors, informing tailored prevention and rehabilitation strategies.

Author (Year)	Country	Design	LOE	Procedure	Key Outcomes
**Screpis et al.** (2023)	Italy	Retrospective propensity-matched cohort 58 patients	III	MOWHTO	In indication-overlap patients >50, MOWHTO produced higher activity levels from 6 months to >4 years than UKA, with similar pain and Lysholm scores. Major complications were rare, though plate removal was common after HTO.
**Coventry et al. (1973)**	USA	Retrospective case series 106 knees	IV	HTO	A classic protocolized series showed proximal tibial valgus osteotomy relieved pain and restored function in most unicompartmental OA knees. Over-correction and meticulous technique were emphasized, with low major complication rates.
**Nagel et al.** (1996)	USA	Retrospective case series 37 knees	IV	LCWHTO	Active men <60 maintained high participation after CW-HTO, though overall activity plateaued or declined slightly over time. Conversion to TKA occurred in ~16%, yet satisfaction remained high.
**Billings et al.** (2000)	USA	Retrospective case series 64 knees	IV	LCWHTO	Using a calibrated cutting guide and rigid fixation, CW-HTO achieved good outcomes with HSS ~94 in unrevised knees and low complication rates. Ten-year survivorship was ~53%, and conversion to TKA was straightforward.
**Noyes et al.** (2000)	USA	Prospective case series 41 knees	IV	HTO	In ACL-deficient varus knees with combined pathology, valgus HTO plus staged/combined ligament reconstruction improved pain, stability, and normalized gait mechanics. Complications were few, supporting alignment correction alongside ligament repair.
**Noyes et al.** (2006)	USA	Prospective case series 114 patients	IV	MOWHTO	A grafted OWHTO technique with early rehabilitation achieved 100% union and preserved tibial slope, with minimal complications. Weight-bearing progressed to full by ~8 weeks, supporting stable fixation and controlled gap geometry.
**Papachristou et al.** (2006)	Greece	Prospective case series 44 knees	IV	HTO	In patients <60 with modest varus, valgus HTO achieved 80% 10-year and 66% 15-year survivorship with improved HSS scores. Alignment drift was small, and complications were infrequent.
**Hui et al.** (2011)	Canada	Retrospective single center long-term case series 455 patients	IV	LCWHTO	Lateral closing-wedge HTO had survivorship of 95% at 5 years and 56% at 15 years, with better longevity in younger, normal-BMI, ACL-deficient patients. Patient satisfaction and OKS were high among survivors.
**Hinterwimmer et al.** (2011)	Germany	Prospective case series 25 patients	IV	MOWHTO	Standardized OWHTO techniques—posterior spreader placement, complete posterior osteotomy, and posterior plate positioning—preserved posterior tibial slope and patellar height. No correlation existed between sagittal changes and the degree of coronal correction.

Author (Year)	Country	Design	LOE	Procedure	Key Outcomes
**Gebhard et al.** (2011)	Germany	Prospective multicenter case series 51 knees	IV	MOWHTO	Computer navigation in OWHTO achieved mean alignment deviations of ~1.5°, with most cases within ±3° of plan. Early technical issues aside, navigation functioned as a reliable intraoperative ruler.
**LaPrade et al.** (2012)	USA	Prospective therapeutic case series 47 knees	IV	MOWHTO	In young to middle-aged adults, OWHTO produced meaningful clinical gains and corrected malalignment with low short-term failure (6%). Posterior tibial slope increased modestly; complications were mainly minor hardware irritation.
**Bonasia et al.** (2014)	Italy	Retrospective case series 99 knees	IV	MOWHTO	OWHTO yielded significant clinical improvement with 5-year survivorship of ~99% and 7.5-year ~76%. Older age and limited postoperative flexion independently predicted worse results; complications were mostly minor and manageable.
**Martin et al.** (2014)	UK	Retrospective case series 323 knees	IV	MOWHTO	In 323 MOWHTOs, severe complications were uncommon (~7%), and most events did not blunt 12–24-month WOMAC gains. Delayed union (12%) was the most frequent issue requiring extended care.
**Yapici et al.** (2020)	Türkiye	Retrospective case series 504 knees	IV	MOWHTO	In 504 MOWHTOs, 10-year survivorship was ~95%, with many minor events but few serious, surgery-requiring complications (~3%). Planned correction was achieved in ~75%, underscoring the importance of alignment accuracy.
**Constantin et al.** (2024)	Australia	Prospective single-centre Long-term case series 95 patients	IV	LCWHTO	Lateral closing-wedge HTO substantially delayed arthroplasty, with 5/10/20-year survivorship of 88%/77%/44% and best longevity in <55 years, BMI<30, and less preoperative pain. Survivors reported high KOOS/WOMAC and satisfaction, supporting careful selection for joint preservation.
**Coventry et al.** (1985)	USA	Narrative review	V	HTO	This current-concepts review recommends HTO for active patients <65 with unicompartmental disease, targeting ~3–5° mechanical valgus. Results decline over time, but osteotomy reliably unloads the medial compartment and postpones arthroplasty when executed precisely.
**Noyes et al.** (2005)	USA	Technical/lab planning method	V	MOWHTO	The 3-Triangle Method provides equations linking desired coronal correction to anterior/posterior gap heights to control tibial slope in OWHTO. Precise anterior gap—about half the posteromedial gap—helps maintain slope, with 1 mm errors altering slope by ~2°.

Author (Year)	Country	Design	LOE	Procedure	Key Outcomes
**Wright et al.** (2005)	USA	Narrative review	V	HTO	This review underscores HTO as a durable, joint-preserving procedure when accurate correction is achieved, with >50% survivorship often beyond 10 years. Technique influences tibial slope and patellar height, and while complications exist, serious events are uncommon.
**Rodner et al.** (2006)	USA	Cadaveric biomechanics 9 cadavers	V	MOWHTO	Anteriorly placed plates in OWHTO increased posterior tibial slope, whereas posterior placement preserved native slope. In ACL-deficient knees, increasing slope shifted peak contact pressures posteriorly, advising posterior plate positioning to avoid posteromedial overload.
**Giffin et al.** (2007)	Canada	Cadaveric biomechanics 10 cadavers	V	MOWHTO	Increasing tibial slope via anterior opening-wedge reduced posterior sag in PCL-deficient knees by shifting tibia anteriorly. While overall laxity was unchanged, anterior translation under load increased, supporting slope-modifying osteotomy for selected PCL-deficient cases.
**Brown et al.** (2012)	USA	Technical planning guide	V	HTO	This technical guide standardizes long-leg planning for valgus-producing HTO, detailing target alignment methods and three-plane correction. It emphasizes accounting for JLCA, controlling tibial slope, and avoiding medial lift-off.
**Smith et al.** (2013)	UK	Narrative review	V	HTO	Reviewing decades of evolution, HTO achieves durable outcomes when targeting the Fujisawa point with modern locking-plate fixation. Complications are technique-specific, and osteotomy remains particularly valuable for younger, active patients and combined procedures.
**Loia et al.** (2016)	Italy	Narrative review	V	MOWHTO	OWHTO planning targets ~62.5% WBL and neutral–3° mechanical valgus, with outcomes best in younger, non-obese patients with good ROM. Technique must control tibial slope and patellar height; mid-term survivorship is strong and TKA conversion is not adversely impacted.
**Mattei et al.** (2017)	Italy	Narrative review	V	LCWHTO	Closing-wedge HTO remains valid in carefully selected, active patients but carries peroneal nerve and fracture risks. Long-term survival declines, conversion to TKA can be challenging, and many centers now favor OWHTO or UKA.
**Khakha et al.** (2021)	UK	Narrative review	V	HTO	Contemporary HTO returns many patients to work and sport and shows acceptable 10-year conversion rates when valgus correction is accurate. Smoking, high BMI, older age, and advanced radiographic disease predict worse outcomes.

Author (Year)	Country	Design	LOE	Procedure	Key Outcomes
**Pullen et al.** (2024)	USA	Narrative review	V	HTO	This review positions HTO as a versatile joint-preserving operation requiring precise three-plane planning to control joint-line obliquity and tibial slope. Long-term survivorship is favorable in selected patients, with technique-specific complication profiles and limited evidence that navigation improves clinical outcomes.
**Griffin et al.** (2007)	Australia	Systematic review 15 studies	II	UKA	UKA and TKA had similar mid-term pain and function, with UKA offering better ROM and possibly fewer thromboembolic events. Compared with HTO, UKA often showed fewer complications; survivorship comparisons were constrained by heterogeneous data.
**Ko et al.** (2015)	South Korea	Systematic review 10 studies	II	UKA	Fixed- and mobile-bearing UKA had similar cumulative reoperation burdens, but failure patterns differed—dislocation/loosening in mobile designs and wear in fixed designs. Mobile designs tended to fail earlier, often within months.
**Kim et al.** (2016)	South Korea	Retrospective complications registry	III	UKA	Among 1,576 UKAs, complications occurred in 5.6%, dominated by mobile-bearing dislocation and component loosening. Many events required conversion to TKA, highlighting design-specific failure modes.
**Cody et al.** (2018)	USA	Retrospective single-surgeon comparative series 569 knees	III	UKA	Outpatient UKA at ambulatory centers was as safe as hospital outpatient care, with comparable 90-day complications and readmissions. Standardized perioperative protocols support safe same-day pathways.
**Ode et al.** (2018)	France	Retrospective matched cohort 120 patients	III	UKA	In patients ≥85, UKA had fewer early medical complications than TKA, with similar mid-term function and survivorship. UKA appears safe and effective for carefully selected very elderly candidates.
**Song et al.** (2009)	South Korea	Retrospective case series 100 knees	IV	UKA	Early minimally invasive Oxford UKA produced strong 2-year clinical improvements with low complication (7%) and revision (3%) rates. Failures clustered in the initial learning curve, emphasizing technique mastery.
**Morris et al.** (2013)	USA	Retrospective series 1000 knees	IV	UKA	In 1,000 consecutive UKAs, 90-day mortality was zero and serious medical events were rare. Secondary procedures were uncommon, supporting UKA's favorable perioperative safety profile.
**Ji et al.** (2014)	South Korea	Retrospective case series 246 knees	IV	UKA	In 246 Oxford UKAs, complications occurred in ~10%, mainly early bearing dislocation and femoral component loosening. Most events required reoperation, though overall patients still improved.

Author (Year)	Country	Design	LOE	Procedure	Key Outcomes
**Riff et al.** (2014)	USA	Narrative review	V	UKA	Modern UKA can achieve 10–15-year survivorship of ~94–98% in expert series, with faster recovery and fewer complications than TKA. Failures are design-specific, and conversion to TKA is usually straightforward with primary components.
**Ashraf et al.** (2020)	Pakistan	Systematic review 12 studies	II	PFO	Systematic review found PFO consistently reduced pain and improved function, with modest valgus shift and medial space widening. Complications were mainly transient neuropathies; higher-quality multicenter trials are needed.
**Sugianto et al.** (2021)	Indonesia	Systematic review & meta-analysis 11 studies	II	PFO	PFO was associated with substantial short-term gains in pain and function and a measurable increase in medial:lateral joint-space ratio. Adverse events were low and mainly transient neuropathies, but evidence quality and heterogeneity limit certainty.
**Liang et al.** (2024)	Singapore	Systematic review 13 studies	II	PFO	Clinical improvement after PFO was substantial across KL-2 to KL-4 cohorts, with modest varus reduction where reported. Complications were predominantly transient nerve symptoms, and overall evidence certainty was moderate to low.
**Jiang et al.** (2025)	China	Systematic review & meta-analysis 21 studies	II	PFO	Pooling 21 studies (including 4 RCTs), PFO produced large improvements in pain, function, and alignment with low rates of mainly transient nerve symptoms. Heterogeneity was substantial and evidence quality low, so long-term randomized comparisons are still needed.
**Irismetov et al.** (2022)	Uzbekistan	Retrospective comparative cohort 152 patients	III	PFO	Adding PFO to arthroscopic debridement improved the proportion of good/very-good outcomes versus debridement alone at ~1 year. No major complications were noted in the PFO cohort.
**Aydın et al.** (2024)	Türkiye	Retrospective cohort 28 patients	III	PFO	After vascularized fibula graft harvest, the donor limb exhibited a small (~1.2°) valgus HKA shift that was statistically significant but likely clinically negligible. Knee and ankle function remained high with low donor-site morbidity.
**Yang et al.** (2015)	China	Retrospective case series 110 knees	IV	PFO	In 110 knees with ~4-year follow-up, PFO improved alignment and function with low but notable transient nerve complications. A small subset progressed to TKA within a year for persistent symptoms.
**Wang et al.** (2017)	China	Retrospective case series 46 patients	IV	PFO	In 46 analyzed patients, PFO yielded significant pain and AKSS improvements and increased medial:lateral joint-space ratio at ~13 months. No postoperative complications were recorded, and weight-bearing was immediate.

Author (Year)	Country	Design	LOE	Procedure	Key Outcomes
**Qin et al.** (2018)	China	Prospective case series 67 knees	IV	PFO	Prospective PFO data showed sustained improvements in HSS, ROM, and pain, with outcomes linked to proximal tibiofibular mechanics and BMI. Neuropathic symptoms were mostly transient, supporting a mechanical unloading mechanism.
**Utomo et al.** (2018)	Indonesia	Prospective case series 15 knees	IV	PFO	In KL IV varus knees, PFO led to measurable valgus correction and large KOOS and OKS improvements at short term. The uncontrolled, small cohort warrants cautious interpretation.
**Subash et al.** (2018)	India	Retrospective case series 30 patients	IV	PFO	In 30 patients, PFO produced marked 2-year improvements in pain and Oxford scores, with medial joint-space widening and modest valgization. Complications were few and transient.
**Elsebaiy et al.** (2019)	Egypt	Retrospective mini case series 10 patients	IV	PFO	In 10 patients, PFO improved KSS and reduced pain within months. Transient dorsum numbness and EHL weakness were frequent, underscoring the small sample and short follow-up.
**Kumar Koduru et al.** (2020)	India	Prospective case series 60 knees	IV	PFO	PFO delivered rapid pain relief (often within 1–2 days) and sustained 2-year improvements in AKSS and medial space ratio. Transient dorsal-foot paresthesias were the main minor complication.
**Yadav et al.** (2020)	India	Prospective case series 40 knees	IV	PFO	A 12-month prospective series showed significant improvements in pain, KSS, and alignment after PFO. No major complications were reported, supporting PFO as a simple, low-risk option.
**Bansal et al.** (2020)	India	Prospective case series 72 knees	IV	PFO	PFO delivered rapid and sustained improvements in pain and KSS over ~18 months, with immediate weight-bearing and short operative times. No significant complications were reported.
**Gupta et al.** (2020)	India	Prospective case series 20 knees	IV	PFO	PFO enabled immediate full weight-bearing and stepwise functional gains through 9 months, with low morbidity. Only one superficial infection was reported.
**Zuber et al.** (2020)	India	Prospective case series 40 patients	IV	PFO	Combining PFO with arthroscopic debridement produced significant 1-year improvements in pain, function, and alignment in KL I–III varus knees. No nerve injuries were reported, and immediate mobilization was routine.
**Ahmed et al.** (2020)	Pakistan	Prospective case series 60 patients	IV	PFO	A 60-patient series showed significant pain reduction, OKS improvement, and medial joint-space widening after PFO. Complications were uncommon and transient, supporting a favorable safety profile.
**Vashisht et al.** (2020)	India	Prospective case series 38 knees	IV	PFO	PFO produced marked early pain relief and functional gains with modest varus correction. Complications were rare and transient, supporting PFO as a safe, minimally invasive option.

Author (Year)	Country	Design	LOE	Procedure	Key Outcomes
**Huda et al.** (2020)	India	Prospective case series 56 knees	IV	PFO	PFO produced strong early symptom relief at 3 months, but benefits diminished by 6–12 months without meaningful coronal realignment. Transient paresthesias were noted; durability appears limited in this series.
**Kakumanu et al.** (2021)	India	Prospective case series 30 knees	IV	PFO	At ≥1 year, PFO significantly reduced pain and increased KSS, with substantial widening of medial joint space and correction of varus alignment. Complications were minimal and transient.
**Ranjan et al.** (2021)	India	Retrospective case series 20 knees	IV	PFO	PFO offered immediate and sustained pain relief through 9 months, with medial:lateral joint-space ratio improvement, particularly in osteoporotic patients. No postoperative complications were observed.
**Bhushan et al.** (2022)	India	Prospective case series 35 knees	IV	PFO	PFO significantly reduced pain and improved WOMAC at short term, with minimal change in ROM. Transient superficial and common peroneal palsies occurred but resolved, emphasizing careful nerve handling.
**Narang et al.** (2022)	India	Prospective case series 32 knees	IV	PFO	A small prospective series reported excellent or good early outcomes in >90% after PFO, with minimal transient nerve symptoms. The procedure appeared effective at 6 months for KL II–III disease.
**Monreal et al.** (2022)	Spain	Retrospective mini series 3 patients	IV	PFO	A three-patient series reported substantial pain relief and medial:lateral joint-space ratio improvement after PFO, without complications. Findings are preliminary due to extremely small sample size.
**Gavrilovski et al.** (2024)	North Macedonia	Retrospective mini case series 14 patients	IV	PFO	In 14 elderly KL III–IV patients, PFO yielded rapid pain relief and functional gains without recorded complications at 6 months. Findings are preliminary due to the small, uncontrolled cohort.
**Karapınar et al.** (2025)	Türkiye	Retrospective case series 230 patients	IV	PFO	In 420 knees, PFO plus percutaneous subchondral drilling produced large short-term improvements in pain, function, and patient-reported health, with modest coronal alignment changes. Complications were infrequent and mainly transient neuropathies, and full weight-bearing began day one.
**Shanmugasundaram et al.** (2019)	India	Narrative review	V	PFO	This narrative review outlines biomechanical rationales for PFO and technique pearls, typically resecting 1–2 cm of fibula 6–10 cm below the fibular head. Early series show prompt pain relief and modest valgization, with mostly transient nerve complications, but high-quality randomized trials are needed.

Author (Year)	Country	Design	LOE	Procedure	Key Outcomes
**Vaish et al.** (2019)	India	Narrative review 7 studies	V	PFO	Across seven mostly Level-IV studies, PFO improved pain and function and allowed early weight-bearing, but evidence quality and follow-up were limited. Transient peroneal-nerve symptoms (~5%) were the main complication, highlighting the importance of a 6–10 cm distal cut.
**Demirkıran et al.** (2020)	Türkiye	Finite-element analysis	V	PFO	Finite-element modeling suggested that adding PFO to medial UKA reduced polyethylene insert contact stresses and shifted load laterally. These simulation results support a plausible biomechanical benefit that warrants clinical testing.
**Wang et al.** (2020)	China	Animal experimental study 40 mice	V	PFO	In a mouse OA model, PFO reduced medial subchondral sclerosis and cartilage degeneration and mitigated malalignment changes. These preclinical findings support a biologic and mechanical basis for symptom improvement.
**Unal et al.** (2021)	Türkiye	Finite-element analysis	V	PFO	Finite-element modeling indicated PFO decreased medial knee contact pressures and balanced tibial plateau load sharing. However, it increased ankle cartilage stresses, suggesting a potential trade-off that needs clinical correlation.
**Kang et al.** (2023)	South Korea	Finite-element analysis	V	PFO	FEA showed PFO consistently lowered medial meniscal and cartilage stresses across proximal, middle, and distal cut levels, especially in greater varus loading. Results support a general unloading mechanism, albeit from single-subject modeling.
**Łuczkiewicz et al.** (2023)	Poland	Hypothesis/concept paper	V	PFO	This concept paper posits that PFO may reduce medial meniscal extrusion by shortening the knee lever arm and lowering the adduction moment. Evidence is indirect, and prospective imaging trials are needed.
**Hüttner et al.** (2024)	Germany	Narrative review/position piece	V	PFO	This position piece explains PFO biomechanics and technique, citing cadaveric evidence of medial pressure reduction and clinical series with acceptable results—even in advanced disease. It advocates careful nerve-sparing technique and calls for multicenter randomized trials before broad adoption.

Table S2. Indications/contraindications & selection signals for HTO, UKA, and PFO for medial-compartment knee OA.

Domain	HTO — Favors (Indications / Selection signals)	HTO — Contra / Caution	UKA — Favors (Indications / Selection signals)	UKA — Contra / Caution	PFO — Candidate profile (Selection signals)	PFO — Contra / Caution
**Age / activity**	Physiologically **<60 y**; active patients aiming to **preserve joint** and delay TKA; realistic with **return-to-sport/work** expectations (RTS ~75–90%) (LOE: reviews/series)	**≥65 y**, low demand; frailty; heavy smokers—↑ nonunion/wound risk (LOE: cohort/review)	**>60 y** (but also 50–65 y when classic anteromedial OA criteria met), **moderate-demand**, desire **faster rehab**; high satisfaction, 10–15 y survival ~**94–98%** in good series (LOE: reviews/series)	High-impact athletes; **gross instability**; diffuse tricompartmental OA; uncontrolled inflammatory disease (LOE: expert/review)	**Middle-aged/older**, **low-to-moderate demand**, symptomatic **varus medial OA** seeking **low-burden, rapid pain relief**; may suit **comorbidity-limited** patients; early full WB typical (LOE: case series/meta-analysis)	Very **young high-demand**, need for durable axis correction; unwilling to accept **short-term data**; occupations at risk with transient **peroneal neuropathy** (LOE: reviews/series)
**BMI / patient factors**	Best with **BMI < 30**; obesity and **smoking/diabetes** → ↑ delayed union & complications (LOE: cohorts/reviews)	**BMI ≫ 30**, heavy smokers, poor bone stock → caution for fixation/graft healing (LOE: reviews)	Ideal **BMI < 30–32**; outcomes sensitive to **surgeon volume/implant choice** (LOE: reviews)	**Morbid obesity**, severe deformity needing large correction; heavy labor expecting deep flexion loads (LOE: reviews)	**Lower BMI** may predict **better pain response**; comorbid patients may benefit from day-case, short anesthesia (LOE: cohort/meta-analysis)	**Obesity with marked tibial varus** if meaningful correction is required (HTO more appropriate) (LOE: expert/series)
**Radiographic OA severity (KL)**	**KL 2–3** (selected **KL4** if goals are only pain relief & delay of TKA and good ROM) (LOE: cohort/review)	**End-stage tricompartmental OA** without joint-preservation goals (LOE: review)	**Anteromedial OA** with preserved lateral & PF compartments; often **KL 3–4** if deformity is correctable (LOE: reviews/RCTs/meta-analyses)	**Bicompartmental / patellofemoral-dominant** disease; **inflammatory** arthropathy (LOE: review)	Works across **KL 1–3** and some **KL4** with **short-term** benefits; outcomes **not strongly dependent on KL grade** in pooled analyses (LOE: pooled analysis)	**Diffuse lateral/PF OA**; **severe deformity** needing large, durable correction (favor HTO/TKA) (LOE: reviews/meta-analysis)
**Alignment (coronal targets / deformity source)**	**Varus 5–15°**; correctable deformity with **tibial-based** component; target **WBL 55–62%** lateral; **MPTA < 95°**, **KJLO < 4–6°** (LOE: technique reviews)	**Valgus OA**, **severe PF pathology** with risk of patella baja after OW-HTO unless tubercle managed; **under-/over-correction** risks (LOE: cohorts/reviews)	**Correctable varus ≤ 10°**, **deformity not primarily tibial**, neutral/physiologic slope; anteromedial pattern with **intact lateral** & PF surfaces (LOE: classic criteria/reviews)	**Uncorrectable deformity**, **extra-articular tibial varus** needing bony correction → prefer HTO (LOE: reviews)	**Mild–moderate varus** (often **<10–12°**), **tibial-sided support** by fibula, **proximal TF-joint morphology** favorable; early valgus "settlement" expected (LOE: series/biomechanics)	**Large deformity** or need for precise, durable correction; **knee hyperlaxity** with major JLCA—HTO preferred (LOE: expert)
**Ligament status / instability**	**ACL/PCL/lateral laxity** (double/triple varus): HTO ± **concomitant/staged ligament reconstruction**; slope modulation as needed (LOE: cohort/biomechanics)	Fixed flexion contracture >**10–15°** without plan for correction; ROM **<90–100°** (LOE: reviews)	**Functionally intact ACL & MCL**; selected **ACL-deficient** cases only with appropriate technique (e.g., slope control/ACLR) (LOE: reviews/systematic review)	**True ACL deficiency** without plan for reconstruction; **multiplanar instability** (LOE: reviews)	**Stable knee**; no gross multi-ligament laxity; **peroneal nerve** course favorable (LOE: series)	**Peroneal neuropathy**, severe valgus knees, extensive lateral compartment collapse (risk of over-correction) (LOE: series)
**Patellofemoral joint (PFJ)**	**Mild PF disease acceptable**; plan for **slope & patellar height** control (OW-HTO → risk of patella baja; consider retrotubercle techniques) (LOE: cohort/technique)	**Severe PF OA** with anterior knee pain—caution with OW-HTO unless addressed (LOE: cohort)	**Minimal PF symptoms**, no lateral/patellar full-thickness loss; **medial** compartment isolated (LOE: classic criteria)	**Advanced PF OA** (anterior knee pain, trochlear wear) (LOE: classic criteria)	**PF status variable**; little high-quality imaging data—assess carefully; avoid if overt PF bone-on-bone (LOE: reviews/series)	**Severe PF OA**, patellar maltracking requiring realignment procedures
**Expected survivorship / durability**	Long-term survival: **~86–100% (5 y)**, **~64–98% (10 y)**, **~44–93% (15 y)**, **~46–85% (20 y)** in selected patients; **age, BMI, Ahlbäck grade, postop valgus** are key predictors (LOE: long-term cohorts)	Deterioration after 10–15 y in older, obese, high-grade OA; under/over-correction worsens durability (LOE: long-term cohorts)	High-volume centres report **~93–98% (10 y)**; **15–25 y** survival often **≥85–95%** with modern implants; failure modes: **bearing dislocation (mobile)**, **loosening/poly wear (fixed)** (LOE: series/reviews)	Young, very active patients may "out-run" polyethylene; severe varus requiring big bony correction (favor HTO)	**Short-term (≤12–24 mo) pain/functional gains**; **modest valgus/JS changes**; **no registry-level durability** (LOE: meta-analyses/series)	**Durability unknown**; avoid when long-horizon axis correction or implant longevity is the primary goal

Table S3. Key complications by procedure.

Complication	HTO (OW/CW)	UKA (mobile or fixed)	PFO
**Hinge/cortical fracture**	**~9%** (OW-HTO; displaced ≥2 mm ~3–6%)	—	—
**Non-union**	**~2%** overall (OW ≈1–2%; CW up to ~4%)	—	—
**Deep infection**	**~0.7%**	**~0.3%**	**<1%** (very rare)
**Nerve injury (procedure-specific)**	**CPN ~3%** (CW-HTO risk); neurovascular overall ~1%	**Bearing-related soft-tissue/MCL injury <1%**	**Sensory numbness ~6%**; **CPN palsy ~2%** (usually transient)
**Loss of correction / implant failure**	**~1%** each (loss of correction / plate failure)	**Bearing dislocation ~2–4%** (mobile) / **loosening ~1–4%** (design-dependent)	**<1–2%** (e.g., fibular fracture or persistent site pain)
**DVT / PE (early)**	**~1–2%** (contemporary series)	**DVT 0.1%, PE ~0%** at 90 days (n=1000)	**≤0.3%** (rare; single DVT in 420-knee series)
**Any complication (composite)**	**~7–10%** (serious ~2–7%)	**~5–10%**	**~6–9%** (mostly transient neuropathies)
**Any reoperation**	**~15%** (mostly plate removal)	**~1–2%** bearing exchange (mobile) within ~1 y; other revisions variable	**Low**; most neuropathies resolve without surgery
**Conversion to arthroplasty**	**~10–12%** by ~3–5 y (OW ~10%; CW ~24%)	**~1–6%** by ~2–5 y (centre/registry-dependent)	**~1–4%** within 1–3 y (series); **long-term unknown**
